# Genetic dissection of NK cell responses

**DOI:** 10.3389/fimmu.2012.00425

**Published:** 2013-01-18

**Authors:** Peter Moussa, Jennifer Marton, Silvia M. Vidal, Nassima Fodil-Cornu

**Affiliations:** ^1^Department of Human Genetics and Department of Microbiology and Immunology, McGill University, Life Sciences ComplexMontreal, QC, Canada; ^2^McGill Centre for the Study of Host Resistance, McGill UniversityMontreal, QC, Canada

**Keywords:** natural killer cells, genetics, GWAS, QTL, NK deficiency, viruses

## Abstract

The association of Natural Killer (NK) cell deficiencies with disease susceptibility has established a central role for NK cells in host defence. In this context, genetic approaches have been pivotal in elucidating and characterizing the molecular mechanisms underlying NK cell function. To this end, homozygosity mapping and linkage analysis in humans have identified mutations that impact NK cell function and cause life-threatening diseases. However, several critical restrictions accompany genetic studies in humans. Studying NK cell pathophysiology in a mouse model has therefore proven a useful tool. The relevance of the mouse model is underscored by the similarities that exist between cell-structure-sensing receptors and the downstream signaling that leads to NK cell activation. In this review, we provide an overview of how human and mouse quantitative trait locis (QTLs) have facilitated the identification of genes that modulate NK cell development, recognition, and killing of target cells.

## Introduction

Progress in the field of genomics over the last few decades has provided the scientific community with invaluable tools that aid in the understanding of genetic diseases. These developments have led to the identification of new techniques, which have been used to study the genetic factors underlying many complex human diseases such as breast cancer, prostate cancer, diabetes, Crohn's disease, and many more (Crawford et al., 2007; Barrett et al., 2008; Imamura and Maeda, 2011; Gudmundsson et al., 2012). Susceptibility to these diseases has been successfully mapped to chromosomal loci in humans via linkage analysis or genome-wide association studies (GWAS). Together, these have increased our understanding of the genetic variants responsible for clinical phenotypes enormously.

Although the techniques outlined above have had an important impact on clinical medicine, they are not without limitations. The most significant limitation occurs in quantitative trait loci (QTL) linkage mapping of disease susceptibility in humans; linkage depends on the careful analysis of segregation of genes from one generation to the next. This requires very large families with clinical and genetic data spanning multiple generations, ultimately limiting the number of families that can be used. Moreover, the resolution is that of segregating chromosomes, which tend to be fairly large regions covering several tens of megabases. GWAS go a long way to address these issues; these studies depend solely on the correlation between phenotype and a specific SNP in a population, thereby increasing resolution to a single base pair. However, soon after its popularity peaked, limitations in GWAS also became apparent. Firstly, despite significant associations between genetic variants and phenotypes, identification of causative variants (be it in promoter or coding regions) remains a major challenge. Secondly, the effect of genetic variants on disease susceptibility may only be apparent in certain environmental conditions. In this case, the presence of a SNP does not necessarily confer a given phenotype, resulting in decreased penetrance. Thirdly, population stratification is a common problem encountered in these studies; a significant SNP may represent a population marker rather than a disease-associated variant. This necessitates the use of larger, more heterogeneous populations. Fourthly, even if larger populations are used, most complex diseases have underlying genetic variants that have low penetrance, which limits the statistical power of these studies. Given the intrinsic limitations associated with human mapping studies, it is apparent that a more controlled genetic model is necessary to compliment discoveries made in humans.

**Figure 1:**
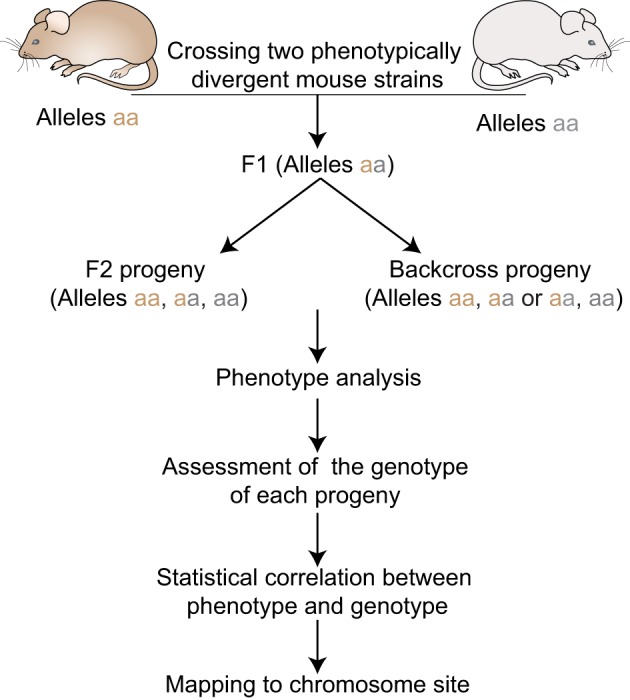
**Quantitative Trait locus mapping in inbred strains.** F1 progeny are generated by crossing two phenotypically divergent strains. F1 mice can be intercrossed to produce F2 progeny or backcrossed to the parental strains to produce N2 progeny. All progenies are phenotyped and genotyped using informative markers across the genome. Statistical phenotype/genotype correlations are carried out to identify genetic loci underlying the given phenotype.

For decades non-human species have been used to model human disease. The mouse has emerged as the most popular mammalian model partly because 99% of mouse genes have homologous counterparts in human. This finding underscores the utility of mouse genetics for identifying genotype–phenotype relationships. To this end, over 450 inbred strains of laboratory and wild-derived mice have been described, providing a panel with ample genetic diversity. Moreover, recent studies have determined that, of the 2.57 billion base pairs that make up the mouse genome, 8.27 million SNPs discriminate the most common laboratory mouse strains (Frazer et al., 2007). This genetic diversity coupled to better control of environmental conditions has facilitated the identification of disease-causing genes using QTL analysis (Figure 1). Moreover, the ability to breed large mouse families quickly has addressed both the statistical power and study design issues encountered in human studies. In this way, QTL mapping approaches in mice have successfully lead to the identification of loci underlying a variety of human traits, including body weight and growth (Corva and Medrano, 2001), obesity (Brockmann and Bevova, 2002), atherosclerosis (Brockmann and Bevova, 2002; Wang et al., 2005), susceptibility to infections (Mayilyan, 2012), cancer (Ewart-Toland et al., 2003), and many aspects of innate immunity, among them Natural Killer (NK) cells (Cook et al., 2004).

In both humans and mice, NK cells are characterized by their natural ability to kill damaged, infected or tumorigenic cells. This recognition of threat is mediated by a repertoir of receptors expressed on the surface of NK cells (Pegram et al., 2011). Although the composition of these receptors differs between humans and mice, the intracellular signaling leading to NK cell effector functions is conserved. Mouse NK cells therefore constitute an appropriate model to study NK cell physiopathology.

In this review we will focus on how studying the genetics of complex human disease in combination with linkage mapping techniques in mouse models has enhanced our understanding of NK cell development, recognition of target cells and cytotoxic effector functions.

## NK cell development

The maturity and number of NK cells depends on a crucial sequence of events which is necessary for them to elicit an efficient and successful immune response. Mature NK cells differ from immature NK cells by the expression of surface receptors and by the strength of their cytotoxic functions. Inefficient maturation usually leads to hyporesponsive cells that cannot clear pathogens efficiently, ultimately leading to more severe infection outcomes. Understanding the maturation process is therefore of pivotal importance and may highlight new therapeutic targets.

Genetic studies have contributed significantly to the understanding of NK cell maturation in both the clinical and research setting. One of the first human deficiencies in NK cells was reported by Eidenschenk et al. (2006). The authors observed low NK cell numbers in the blood of four supposedly unrelated patients of Irish nomadic descent. Other symptoms included EBV driven lymphoma and viral susceptibilities which seemed to be a direct result of low NK cell activity. Given the high incidence of inbreeding in the study population, linkage mapping seemed an appropriate tool to dissect the genetic contribution to this phenotype. Homozygosity mapping was conducted in 13 family members. A single significant QTL (LOD: 4.51) linked NK deficiency to a locus on chr 8 (8p12-q12.2). A common haplotype was observed in all four patients (Eidenschenk et al., 2006).

The gene responsible for this deficiency was not found until 4 years later, when additional patients were considered in an analysis by Gineau et al. (2012). These individuals displayed similar phenotypes and allelic profiles to the patients in the initial cohort. Remarkably, linkage analysis using all individuals identified a single, highly significant QTL (LOD: 6.45) localizing to the same region on chr 8. Sequence analysis discovered a specific frame shift mutation in MCM4, a component of the mini chromosome maintenance complex, which is important for DNA replication initiation and elongation. This mutation introduced a premature stop codon thereby truncating the wild-type protein (863 amino acids) to 27 amino acids. Further analysis of this mutant protein showed that it could still bind to the rest of the mini chromosome maintenance complex and to chromatin. However, the complex could not control the prevention of re-replication in normal fashion. This inefficient control led to multiple DNA aberrations and breaks, which could be rescued by expressing wild-type MCM4 in these cells. Interestingly, other myeloid and lymphoid cell subpopulations were normal; only NK cells were affected.

Concurrent with the low NK cell numbers, there was selective loss in the CD56^dim^ (more mature) subset of NK cells as opposed to the CD56^bright^ (more immature) subset. Terminal differentiation usually occurs after NK cells have achieved CD56^dim^ status and is characterized by the appearance of CD57, the loss of CD94, decreased proliferation and increased cytolytic activity. Surprisingly very few NK cells from these patients showed terminal maturation, thereby stressing the importance of MCM4 in NK cell maturation. Moreover, the CD56^bright^ subset of cells showed spontaneous apoptosis which was rescued upon IL2 and IL15 stimulation. Their proliferation capacity, however, was much less than control cells, even upon stimulation. Conversely, although CD56^dim^ cells also showed excessive spontaneous apoptosis, which was not rescued upon IL2 and IL15 stimulation, they still proliferated as well as control cells. Altogether, these results suggested that the low levels of CD56^dim^ cells likely results due to a failure of the CD56^bright^ cells to proliferate due to DNA breaks (Gineau et al., 2012).

**Figure 2:**
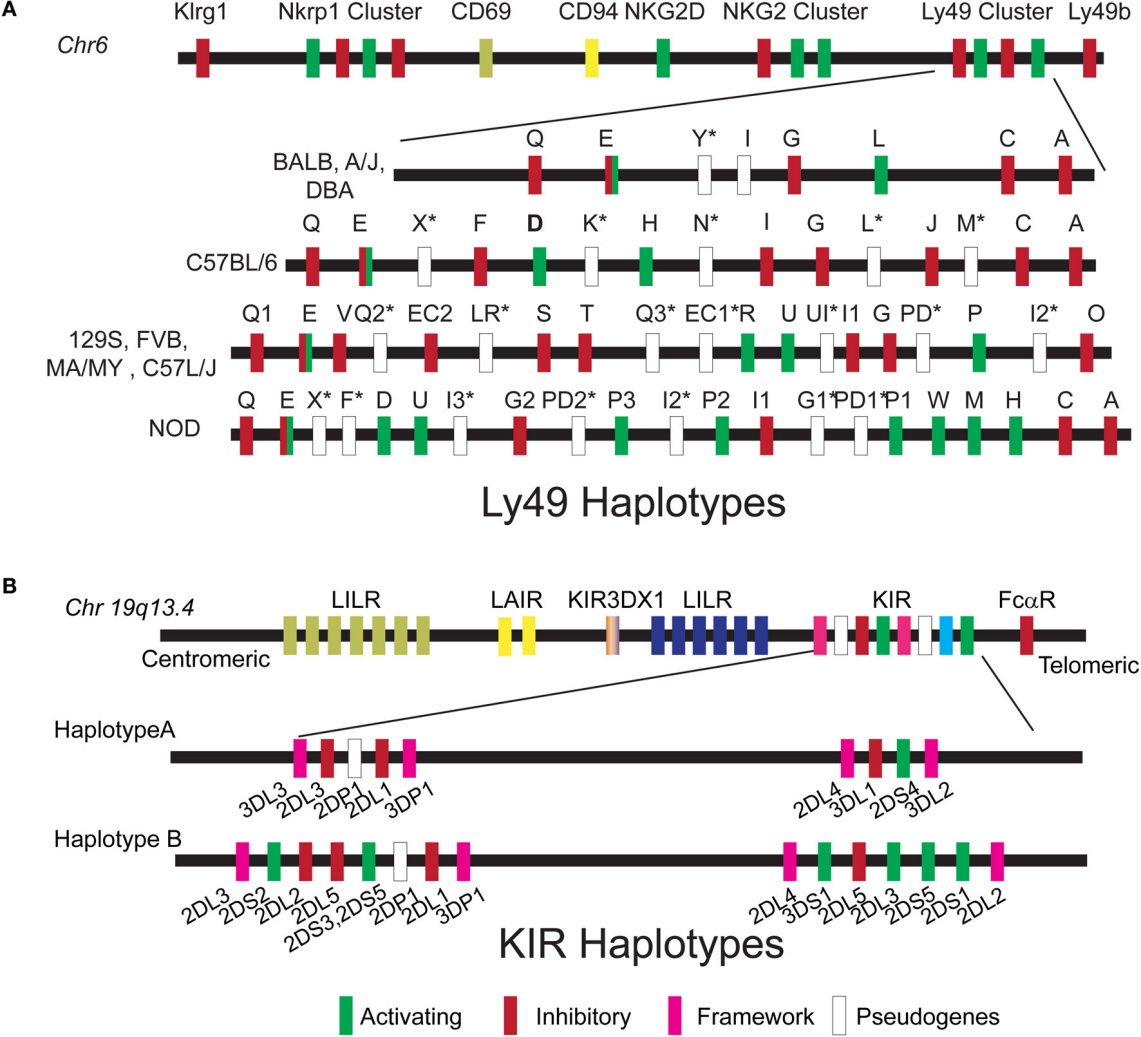
**Ly49 and KIR haplotypes. (A)** The *Ly49* gene cluster is located on chr 6 within the Natural Killer gene Complex. Schematic organization of the *Ly49* haplotype is described for the inbred mouse strains that were used to map NK cell function by QTL. **(B)** The *KIR* gene cluster is located on chromosome 19q13.4 within the leukocyte receptor complex. Two basic haplotypes A and B have been defined, varying in the number of activating KIR genes. Haplotype A is uniform in terms of gene content and is composed of five inhibitory genes (*KIR2DL1*, *2DL3*, *3DL1*, *3DL2*, and *3DL3*), a single activating gene *KIR2DS4*, and *KIR3DL4* which may have both inhibitory and activating capacities. B haplotypes contain a variable combination of activating (*2DS1*, *2DS2*, *2DS3*, *2DS5*, *3DS1*, and *2DS4*) and inhibitory genes (*KIR2DL5A/B* and *KIR2DL2*).

Spontaneous defects in NK cell development have also been observed in mice. Non-obese diabetic (NOD) mice exhibit an NK cell deficiency characterized by a reduced ability to kill YAC-1 target cells *in vitro*, likely resulting from reduced NKG2D levels (Ogasawara et al., 2003). In addition, NK cells from NOD mice have a non-typical repertoire of NK receptors (Figure 2) that affect NK cell function and survival (Belanger et al., 2008). To explore this trait, Suwanai et al. examined the genetic control of NK cell rejection of MHC-I deficient cells in NOD mice compared to B6.H-2^g7^ (a mouse strain carrying the NOD derived H2 locus) (Suwanai et al., 2010). In this study, NOD and B6.H-2^g7^ mice had similar NK cell numbers and maturity but the cytotoxic capacity of B6.H-2^g7^ mice was 2–3-fold higher than that of NOD mice. Linkage analysis of the F2 progeny using either NK cell number or rejection capacity as mapping traits revealed a dominant locus on chr 8 with epistatic effects of chr1, 6, and 8. A positional cloning approach was utilized to better define the genetic region responsible for NK cell deficiency. Subcongenic mice were generated which narrowed the chr 8 QTL to a 2 Mbp region between 83 and 85 Mbp. This locus contained 9 genes, including the one encoding IL15. Genome-wide expression analyses in the spleenocytes of B6.H-2^g7^ and NOD mice revealed that IL15 was differentially expressed. As IL15 is an important cytokine for NK cell maturation, survival, and function (Ma et al., 2006), it became the principal candidate in this study. The defect in IL15 expression was not NK intrinsic given that IL15 expression in macrophages and dendritic cells was also affected. Furthermore, supplementation of NOD mice with soluble IL15RαFc rescued NK cell deficiency and perforin secretion, confirming that a defect in IL15 gene expression is a major contributor to the NK cell defects observed in NOD mice.

Collectively, studies in both humans and the mouse model have been pivotal in elucidating the genes involved in NK cell maturation and development. The human studies were crucial in identifying a mutation which inhibits NK cells from reaching their peak maturity and therefore hinders their antiviral response. The mouse studies functioned as a proof of principle, emphasizing the importance of IL15 in NK cell maturation and providing evidence that mouse models can be efficiently used in order to decipher inefficiencies in NK cell development.

## NK cell recognition

NK cells were first characterized by their ability to efficiently kill cells lacking major histocompatibility complex (MHC) (Stern et al., 1980). *In vivo* rejection of radiolabeled tumor cell lines either expressing MHC-I or not (RMA and RNA/s, respectively) suggested that the recognition of MHC molecules may constitute an inhibitory signal in the NK—target interaction (Ljunggren and Karre, 1985). Thus, the decision to kill depends on the equilibrium between opposing activating and inhibitory signals that arise from activating and inhibitory receptors expressed on the surface of NK cells. NK cell receptors for classical MHC class I molecules are encoded by the killer Ig-like receptor (KIR) multigene family in humans and the C-type lectin-like genes family (Ly49) in mice. These receptors are polygenic, polymorphic, and stochastically expressed (Valiante et al., 1997; Anderson, 2006; Brown and Scalzo, 2008). The *Ly49* gene family resides within the NKC on chr 6 and different NKC haplotypes have been described in inbred mice (Carlyle et al., 2008; Higuchi et al., 2010) (Figure 2A). The *KIR* genes are located on chr 19 and two major classes of *KIR* haplotypes have been identified: Haplotype A that is predominately inhibitory and haplotype B that contains various combinations of activating and inhibitory *KIR* genes (Figure 2B). In addition to this haplotype diversity, multiple alleles exist for each *KIR* gene. Each can vary in expression level and functional capacity. Here we will discuss how vast genetic differences in the human *KIR* and mouse *Ly49* genes families have been successfully used to dissect different NK cell responses and identify loci that control these responses in the context of viral infections.

It has been difficult to study the direct effect of individual KIR receptor(s) recognition of target cells on disease outcome; the high diversity of KIRs and the lack of specific tools distinguishing between the different KIR alleles have hindered this progress. Given the interaction between KIR and HLA-class I molecules, the majority of studies are based on genetic association of KIRs in the context of their HLA-class I ligands (for review see Khakoo and Carrington, 2006). In acquired immune deficiency syndrome (AIDS), the first genetic association described was between HIV disease progression and HLA-B alleles. This association was shown in a small cohort of seropositive patients wherein a significant association between homozygosity for alleles that share the HLA-Bw4 epitope and the control of HIV-1 viremia was observed (Flores-Villanueva et al., 2001). Subsequent HLA-class I typing and KIR genotyping determined that the activating KIR allele KIR3DS1, in combination with HLA-B Bw4-80Ile, is associated with delayed progression to AIDS (Martin et al., 2002). Although a physical interaction between KIR3DS1 and HLA-Bw4-80I has not yet been confirmed, functional data has shown that NK cells expressing KIR3DS1 mount a more potent response to HIV-infected Bw4-80I CD4+ T cells (Alter et al., 2007). Moreover, NK cells expressing KIR3DS1 were more responsive to HLA-class I negative target cells. This responsiveness was more profound among individuals that coexpressed KIR3DS1 and Bw4-80I (Long et al., 2008). These data suggest that the activating KIR might recognize a viral component in the context of HLA-Bw4-80I, leading to NK cytotoxic effects. This is reminiscent of the interaction between Ly49P/H2Dk and the viral protein mo4/gp34 during mouse cytomegalovirus (MCMV) infection which will be further discussed below (Desrosiers et al., 2005).

HLA-Bw4 molecules are not only ligands for KIR3DS1 but also for the many inhibitory KIR3DL1 alleles (Gumperz et al., 1995; Carr et al., 2005). It is interesting to note that other genetic association studies have demonstrated that some KIR3DL1 alleles are associated with slower HIV-1 disease progression, when coexpressed with Bw4-80I alleles (Martin et al., 2007). In this case, KIR3DL1-mediated protection in the context of high expression might be the consequence of better education during NK cell development, leading to increased NK cell functional competence as previously described (Fernandez et al., 2005; Kim et al., 2005, 2008; Anfossi et al., 2006).

A protective effect of KIR3DS1 and HLA-Bw4 was also found in studies examining the association of KIR/HLA and hepatitis C virus (HCV) (Khakoo et al., 2004). Resolution of low-dose HCV infection was associated with a weak interaction between KIR2DL3-HLA-C1. The weak inhibitory signal may result in a greater propensity toward activating signals, thereby leading to improved NK cell responses and increased protection as was shown in a mouse model of MCMV infection (Fodil-Cornu et al., 2011). Conversely, the KIR2DL3-HLA-C1 interaction was significantly associated with susceptibility to cerebral malaria in a case-control study of patients from Thailand (Hirayasu et al., 2012). To address whether natural selection due to cerebral malaria might act on both KIR2DL3 and HLA-C1, the KIR-HLA association was analysed in malaria patients from several worldwide populations (Single et al., 2007; Hirayasu et al., 2012). Interestingly, in highly endemic populations, the frequency of KIR2DL3 and HLA-C1 was significantly decreased, presumably due to a fatal interaction between KIR2DL-HLA-C1 during cerebral malaria infection (Hirayasu et al., 2012). The importance of the KIR/HLA interaction for infection outcome was also highlighted in a patient whose NK cells expressed the inhibitory receptor KIR2DL1. KIR2DL1 interacts strongly with HLA-C proteins. In this particular patient, the interaction resulted in extreme NK cell inhibition and in recurrent CMV infections (Gazit et al., 2004). The examples outlined above suggests that inhibitory NK cell interaction through KIR or Ly49 receptors are important in determining NK cell recognition of target cells and anti-pathological responses.

**Figure 3:**
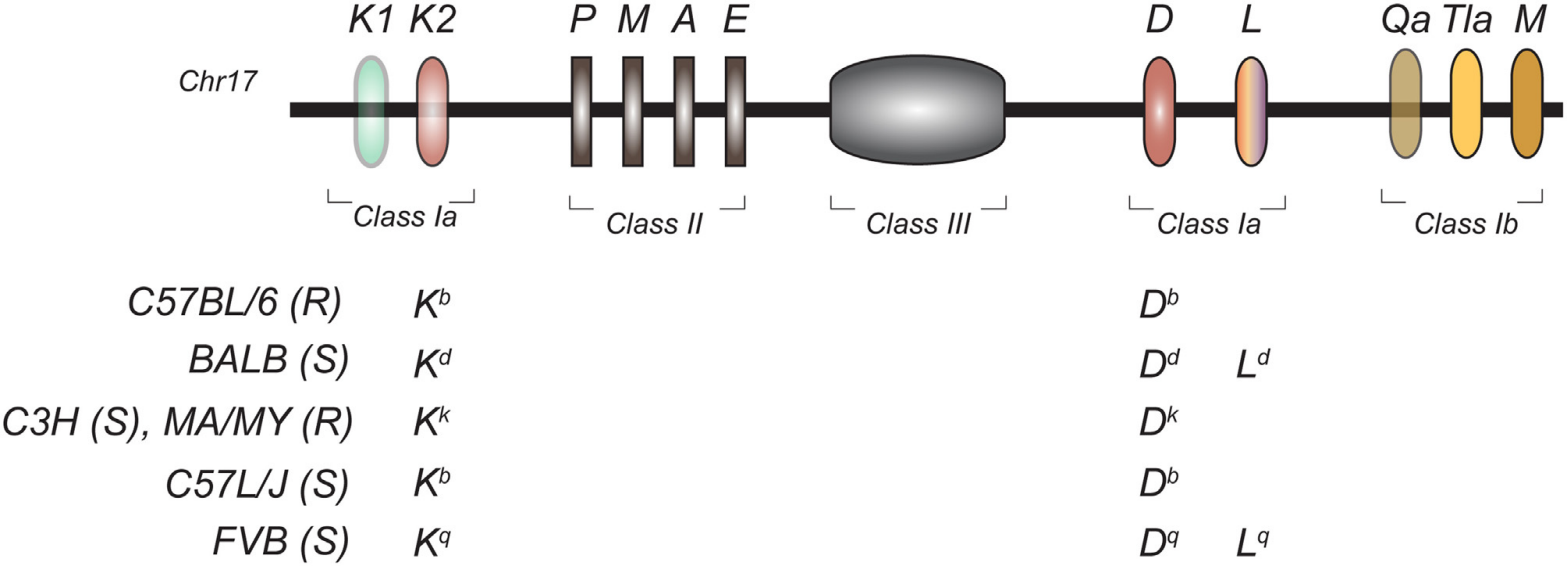
**Genomic organization of the mouse MHC region in inbred mouse strains.** The H2 is located on chromosome 17 and encodes for classical class I (Ia) and non-classical class I (Ib), class II, and class III molecules. The MHC class Ia haplotypes of the inbred mouse strains used for MCMV QTL mapping is shown. (S), Susceptible to MCMV; (R), Resistant to MCMV.

Although human studies have successfully identified genetic associations between NK cells and clinical disease, direct evidence supporting the role of NK cell recognition in the outcome of infection stems from studies in the mouse model. Early studies investigating the genetic determinism of lethal MCMV infection established that different strains of mice respond differently to MCMV challenge (Selgrade and Osborn, 1974). Later, it was reported that the outcome of MCMV infection was dependent both on H2 genes in the MHC (Figure 3) (Chalmer et al., 1977) as well as on non-H-2 linked genes (Grundy et al., 1981). Non-H-2 determinants included the *Cmv1* locus in C57BL/6 mice (Scalzo et al., 1990) and *Cmv3* in MA/My (Desrosiers et al., 2005). Both loci mapped to the NKC.

**Figure 4:**
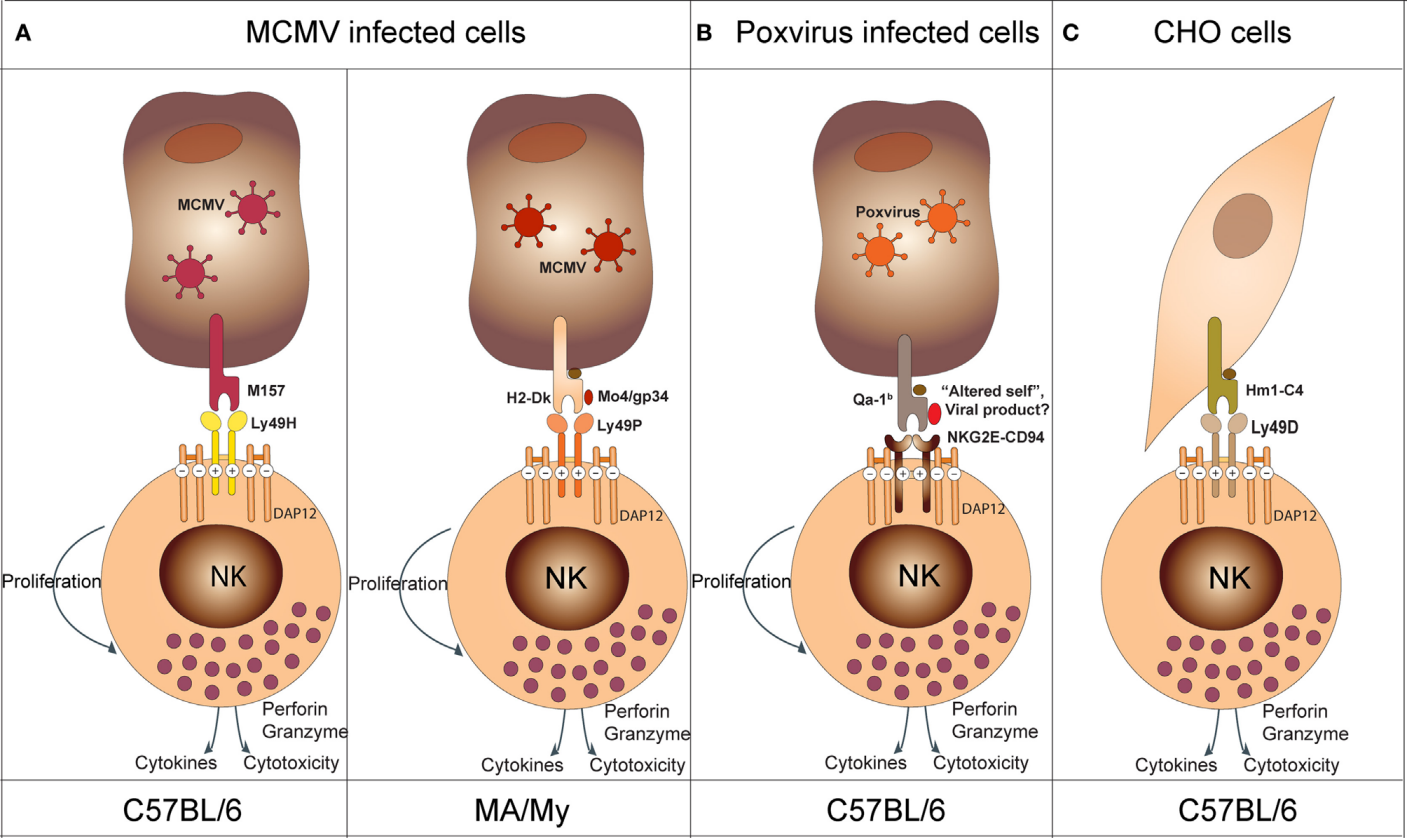
**Model depicting NK cell recognition of target cells. (A)** In the context of MCMV infection, Ly49H (in C57BL/6 mice) recognizes the viral product M157 and Ly49P (in MA/My mice) recognizes the viral product M04/gp34 in association with H2-D^k^. **(B)** In the context of poxvirus infection, NKG2E-CD94 recognizes an “altered self” or a viral product associated with the non-classical Qa-1^b^ molecule (NKG2E-CD94 activates NK cells in synergy with NKG2D upon NKG2D—ligand interaction; see text). **(C)** In the context of xenogeneic cell recognition, Ly49D recognizes the hamster Hm1-C4 MHC class I molecule expressed on CHO cells.

Years of breeding, physical mapping, and positional cloning were required to definitively identify the gene underlying the *Cmv1* locus: Ly49H. Ly49H is an NK cell activating receptor required to establish resistance to MCMV infection (Scalzo et al., 1990, 1992; Depatie et al., 1997, 1999, 2000; Forbes et al., 1997; Lee et al., 2001). Corroborating evidence for the pivotal role of Ly49H was also seen from transgenic, knock-out and congenic mouse studies (Sjolin et al., 2002; Lee et al., 2003; Cheng et al., 2008; Fodil-Cornu et al., 2008). Functional assays determined that the m157 glycoprotein, an MCMV viral product, was the ligand for Ly49H (Figure 4A) (Arase et al., 2002; Voigt et al., 2003). This was further validated by *in vivo* experiments demonstrating increased virulence of an MCMV strain lacking m157 in C57BL6 mice (Bubic et al., 2004). Moreover, these studies provided unique tools to better dissect and define NK cell specific functions. These tools were used to show the following: (1) NK cells have a regulatory function at later times post infection and this function is sustained through m157 stimulation of Ly49H (Lee et al., 2009), (2) in perforin knock-out mice, IL-10 production by NK cells following Ly49H stimulation confers a survival advantage compared to Ly49H^−/−^ mice during lethal MCMV infection (Lee et al., 2009) (3) experienced Ly49H+NK cells during viral infection acquire a memory phenotype which renders them more protective than naïve NK cells (Sun et al., 2009).

The Ly49H-m157 receptor-ligand pair remains the only example of an NK cell activating receptor binding to a private viral protein. The genetic characterization of the *Cmv3* resistance locus in MA/MY mice revealed a distinct mechanism of MCMV-infected cell recognition (Desrosiers et al., 2005). In these mice, *Cmv3*-mediated resistance was strictly dependent on the H2 haplotype, H2k. The generation of a panel of congenic mice and transgenic mice confirmed the importance of both MA/My NKC and H2-Dk in conferring MCMV resistance (Xie et al., 2010; Fodil-Cornu et al., 2011). The recognition of MCMV-infected cells by MA/My NK cells depended on the presence of the H2-Dk molecule in combination with the MCMV encoded protein, M04/gp34 (Figure 4A) (Kielczewska et al., 2009). Moreover, Ly49 activating receptors providing H2-dependent m04-dependent recognition of MCMV-infected cells seems to be a recurrent mechanism in different inbred strains. However, the nature of the receptor and H2 molecule can differ. For example, activating Ly49L from BALB/c mice can detect MCMV-infected cells in the context of H2k and H2f haplotypes (Pyzik et al., 2011). In this case, BALB/c mice have both NK cell and H2k-dependent control of viral spread as compared to H2d or H2b animals. This advantage, however, is abrogated during infection with Δm04 MCMV. Another example has been observed in PWK/Pas mice. NK cells from these mice express Ly49D2 which recognizes MCMV-infected cells in the context of H2k. The protective effect of this interaction, however, remains to be demonstrated *in vivo*. Altogether, these data suggest that MCMV provided an important evolutionary pressure for the diversification of activating Ly49 receptors. Moreover, they highlighted two distinct mechanisms of MCMV recognition by NK cells: the direct Ly49H-dependent recognition of “non-self” product (m157), and the indirect Ly49P, L and D2-dependent recognition of “altered self” (H2-Dk/M04).

The first study implicating NK cells in the control of Ectromelia virus (mousepox) was reported more than 20 years ago. In this study, NK cell depletion reverted ectromelia outcome in resistant B6 mice (Jacoby et al., 1989). Subsequently, several informative crosses identified four major QTLs controlling the outcome of lethal mousepox infection (Brownstein et al., 1991, 1992; Brownstein and Gras, 1995; Delano and Brownstein, 1995). One among them, Rmp1, was linked to the NKC (Delano and Brownstein, 1995). Studies looking to provide mechanistic insight demonstrated that NK cells were important both for the inhibition of early viral replication and to promote strong T-cell responses (Parker et al., 2007; Fang et al., 2008). The search for relevant activating NK receptor(s) identified NKG2D (Fang et al., 2008). This receptor likely facilitates NK cell-dependent killing of infected cells that express NKG2D ligands. Interestingly, however, the blocking of NKG2D receptor induced only 50% mortality in resistant strains of mice challenged with ectromelia. This suggested the involvement of other receptors. Further studies have implicated the CD94-NKG2E receptors in susceptibility to ectromelia (Fang et al., 2011) (Figure 4B). In this model, synergistic effects of NKG2D and CD94-NKG2A are required to confer protection against Ectromelia. The precise molecular mechanisms, however, remain elusive.

Activating Ly49 receptors can be also involved in NK cell lysis of certain xenogeneic cells. The original observation was that B6 IL2–activated NK (LAK) cells or fresh isolated NK cells could kill Chinese hamster ovary cells (CHO) more efficiently than their BALB/c counterparts (Idris et al., 1998). The genetic locus underlying this trait, *chok*, was also shown to control tumor elimination *in vivo*. An Ly49D neutralizing antibody could decrease the cytotoxic capacity of B6 mice to BALB/c levels. This indicated that Ly49D is involved in the recognition of CHO cells (Idris et al., 1999). Ly49D also specifically stimulated the natural killing of lymphoblast targets derived from wild-type and several MHC-congenic rats. Killing occurred in rats possessing the RT1lv1 and RT1l MHC haplotypes but not others (RT1c, RT1u, RT1av1, or RT1n). Given the ability of Ly-49D to mediate cytotoxicity against xenogeneic cells, it was suggested that Ly-49D may recognize xenogeneic MHC-encoded ligands. As expected, engraftment of RMA transfected with Hm1-C4 MHC class I molecule from hamsters showed that Hm1-C4 is the ligand of Ly49D normally expressed by CHO cells (Merck et al., 2009) (Figure 4C). The specificity of Ly49D to a xenogeneic ligand could be the result of an evolutionary mechanism developed to protect the host from a viral protein expressed on infected cells, much like Ly49H with m157. This would imply that Ly49D activation by xenogeneic cells might have arisen due to cross reactivity with similar viral and host molecules expressed on cell surfaces.

Altogether, the genetic studies demonstrated that activating receptors are implicated in the recognition of many molecular determinants that are induced by infection or stress. Moreover, the MCMV and ectromelia models nicely demonstrate that viruses exert an *evolutionary pressure* that drives new mechanisms of resistance to viral infections.

## NK cell killing

Upon activation, NK cell effector functions include both cytotoxicity and cytokine production. Activation of NK cells requires the presence of pro-inflammatory cytokines, such as type I interferons (IFN), IL12, 15, 18, and 21 as well as well as the engagement of cell surface receptors (Biron et al., 1999). The primary cytotoxic mechanisms used by NK cells to kill target cells consist of perforin, a membrane disturbing protein, and granzymes, a family of serine proteases, that together induce apoptosis (Lowin et al., 1995). NK cells can also trigger target cell apoptosis through members of the tumor necrosis factor (TNF) superfamily such as the death receptor-ligands FasL and TRAIL (Smyth et al., 2005).

**Figure 5:**
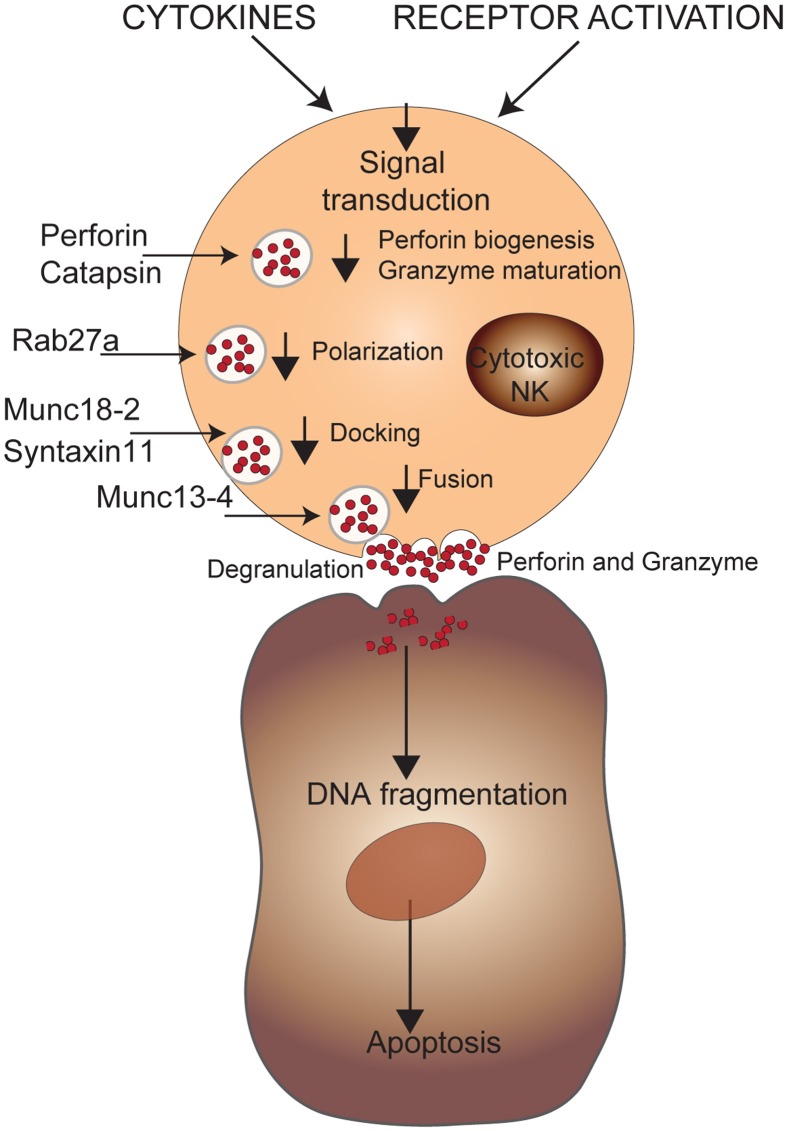
**Genetic defects in NK cell cytotoxicity.** NK cell effector functions are triggered through activating receptors and/or cytokines. A cascade of intracellular events will induce the biogenesis of perforin and maturation of granzyme B that will be secreted via cytotoxic granules leading to apoptosis of target cells via DNA fragmentation. The mutations affecting maturation of the cytotoxic granules or their transport to the cell membrane are shown.

The importance of perforin in NK cell cytotoxicity was highlighted by analyzing the genetic defect underlying a special human disorder of immune dysregulation known as familial haemophagocytic lymphohistiocytosis (FHL). In this disease, cytotoxic T and NK cell activity was consistently low or absent (Perez et al., 1984; Arico et al., 1988; Eife et al., 1989; Egeler et al., 1996). A genome-wide linkage search for the FHL locus was first conducted in 17 families, and identified the FHL2 locus on the proximal region of chromosome 10q21-22 (Dufourcq-Lagelouse et al., 1999). Further studies showed that this locus encodes for perforin and confirmed the mutation in eight unrelated patients with FHL (Stepp et al., 1999). Functional studies demonstrated decreased cytotoxicity and low levels or absence of perforin in lymphocytes derived from FHL patients (Stepp et al., 1999). In other genome-wide screens of FHL families that excluded the perforin mutations (FHL2 locus), two other susceptibility loci were found. The first locus, FHL3 was linked to chr 17q25 (Feldmann et al., 2003). Sequence analysis and protein prediction within this region identified the UNC13D gene encoding for Munc13-4 that was found mutated in the affected patients (Feldmann et al., 2003). Functional studies showed that lytic granules in Munc13-4-deficient cells traffic to the cell membrane and dock, but are incapable of fusing. This data demonstrated that Munc13-4 is essential in priming the lytic granule for membrane fusion and exocytosis (Feldmann et al., 2003). Further molecular analysis showed that the interaction between Munc13-4 and Rab27a [which is associated with the immune disorder Griscelli syndrome type 2 (GS2)] is crucial for degranulation and cytotoxicity, and that mutations in Munc13-4 abrogate this interaction (Neeft et al., 2005; Zur Stadt et al., 2006; Menager et al., 2007; Wood et al., 2009). Interestingly, mutations in Munc13-4 in the mouse causes hemophagocytic lymphohistiocytosis like syndrome only upon lymphocytic choriomeningitis virus (LCMV) infection (Crozat et al., 2007). This observation is consistent with the hypothesis that expression of FHL phenotype is dependent on an infectious trigger (Fisman, 2000). The second locus, FHL4 was found in a genome-wide homozygosity analysis in a large consanguineous FHL kindred of Kurdish descent (Zur Stadt et al., 2005). This QTL localized to a 10cM region of chr 6q24. Analysis of candidate genes within the region identified several different mutations in the Syntaxin 11 (STX11) gene in all affected patients (Zur Stadt et al., 2005). STX11 is a soluble N-ethylmaleimide sensitive factor attachment protein receptor (SNARE) protein that functions to facilitate Rab27a lytic granule adherence to the inside surface of the NK cell membrane (Dabrazhynetskaya et al., 2012). Both Munc13-4 and STX11 mutations have a very similar outcome which is lacking the ability to secrete cytotoxic substances like perforin and granzyme to the target cell interface. Finally, the generation of Stx11^(−/−)^ mice demonstrated that the requirement of STX11 is for NK and CD8(+) T-cell cytotoxicity as well as for neutrophil degranulation (D'Orlando et al., 2012). The last FHL locus, FHL5 was identified using high-resolution SNP genotyping in eight unrelated FHL patients from consanguineous families (in which *PRF1*, *UNC13D*, or STX11 genes were intact) and mapped to a 1 Mb region on chromosome 19p (Zur Stadt et al., 2009). The causative gene was shown to be *STXBP2* encoding syntaxin binding protein 2 (Munc18-2), a protein involved in regulation of intracellular trafficking and control of SNARE complex (Zur Stadt et al., 2009). In another study, similar mutations in Munc18-2 demonstrated that STXBP2 is required at a late step of the secretory pathway, specifically for the release of cytotoxic granules by binding STX11 (Figure 5) (Cote et al., 2009).

Another genetic disorder involving defects in NK cell cytolytic activity was first described by Papillion and Lefevre in 1924. Known as Papillion–Lefevre syndrome (PLS), it is characterized by palmoplantar keratosis, as well as premature loss of deciduous and permanent teeth (Hart et al., 1998). In the late 1990's, the cause of this keratosis was unknown; however, it was generally believed that an epithelial defect rendered tissues susceptible to infection, leading to excess periodontal destruction (Preus, 1988; Soskolne et al., 1996). To address this anomaly, Firatli et al. conducted a study involving 10 families, totalling 44 people, of which 14 were PLS patients (Hart et al., 1998). Pedigree analysis revealed that this was an inherited autosomal recessive disease. Homozygosity linkage mapping was used on the PLS patients and identified a significant peak on chr 11q14-21 (Hart et al., 1998). Using finer mapping and haplotype reconstruction, the candidate region was narrowed down to 4-5cM interval. A year later, a study by Toomes et al. narrowed this region down even further to 1.2cM. The interval contained Cathepsin C which was later shown to be the gene responsible for PLS (Toomes et al., 1999). They showed the cDNA sequence was composed of 7 exons and 6 introns, identical to the previously described mouse gene. They also confirmed the protein defect in PLS patients via functional assays (Pham et al., 1997). The same year, studies in mice characterized the function of Cathepsin C, and found it to be involved in the cleavage of pro-granzyme B and A to their functional isoforms in mouse LAK cells (Figure 5) (Pham and Ley, 1999). Interestingly, a later study by Meade et al. found that, unlike the mouse model, PLS patient LAK cells were normal. However, unstimulated PLS NK cells displayed a drastic deficiency in granzyme B activity when compared to controls (Meade et al., 2006). Moreover, western blots showed that granzyme B in PLS patients was larger than in controls, proving that they did not get processed to their active forms. When cathepsin C activity was monitored, it appeared that its activity was low in both IL2 stimulated and unstimulated PLS NK cells. This could be due to another redundant gene that takes over the function of cathepsin when stimulated with IL2 or that, unlike the mouse model, human NK cells did not need cathepsin C for granzyme B processing/activity. The granzyme B deficiency in unstimulated PLS NK cells was correlated to their low ability to kill target cells in an independent perforin manner. Thus, this study showed that granzyme B is present in PLS patients, but due to a processing disorder that is independent of cathepsin C, is inactive. Cathepsin C, however, is probably still the causative mutation of PLS since it is also involved in the processing of other granzymes and has been shown in many independent mapping studies to be linked to PLS (Toomes et al., 1999). Deciphering the genetic etiology of PLS exemplifies how mouse studies can provide mechanistic insight into human diseases, but should be taken with a grain of salt.

## Concluding remarks

The mouse model has proven invaluable for the dissection of factors that affect immune cell function. In particular, these models have greatly advanced our understanding of NK cell metabolism, most notably in the context of infectious disease. They have provided insight that was otherwise inaccessible due to the design and technological restrictions associated with human studies. Although many of these restrictions have been addressed with the advent of rapid and accurate next generation sequencing platforms, mouse models remain relevant. Their continued utility is particularly evident for the study of HLA/KIR interactions. The massive variation present between individuals makes studying these nearly impossible in humans. Newly developed humanized mice, however, can be used to dissect these interactions and decipher their effect on various human conditions (be it in response to infection or in the context of autoimmune disease). The relevance of humanized mice for studying human diseases is further emphasized by the fact that some can be infected with human pathogens such as HCMV. Aside from humanized mice, large-scale ENU projects have identified many genes relevant for human disease. The main advantage to these studies is that mice usually display a strong phenotype upon mutation and this can be studied independently of any genetic background effect. Although the genes identified in ENU screens do not always generate a comparable phenotype in humans, they often alter the function of a pathway that is common to humans. In this way, these studies remain informative for human disease as they can provide mechanistic insight. Thus, although new technologies have greatly enhanced our discovery potential in the realm of genetics, the mouse model will continue to serve as an important compliment. Together they will work to further our knowledge of genetics and its role in human disease.

### Conflict of interest statement

The authors declare that the research was conducted in the absence of any commercial or financial relationships that could be construed as a potential conflict of interest.
